# Oxidative Stress and Related Biomarkers in Gilbert’s Syndrome: A Secondary Analysis of Two Case-Control Studies

**DOI:** 10.3390/antiox10091474

**Published:** 2021-09-15

**Authors:** Karl-Heinz Wagner, Nazlisadat Seyed Khoei, Claudia Anna Hana, Daniel Doberer, Rodrig Marculescu, Andrew Cameron Bulmer, Marlies Hörmann-Wallner, Christine Mölzer

**Affiliations:** 1 Department of Nutritional Sciences, Faculty of Life Sciences, University of Vienna, 1090 Vienna, Austria; Claudia.hana@univie.ac.at; 2 Research Platform Active Ageing, University of Vienna, 1090 Vienna, Austria; nazlisadat.seyedkhoei@univie.ac.at; 3 Department of Clinical Pharmacology, Medical University of Vienna, Vienna General Hospital, 1090 Vienna, Austria; daniel.doberer@meduniwien.ac.at; 4 Clinical Institute of Laboratory Medicine, Medical University of Vienna, Vienna General Hospital, 1090 Vienna, Austria; rodrig.marculescu@meduniwien.ac.at; 5 Menzies Health Institute Queensland, School of Medical Science, Griffith University, Brisbane, QLD 4222, Australia; a.bulmer@griffith.edu.au; 6 Institute for Dietetics and Nutrition, University of Applied Sciences FH JOANNEUM, 8020 Graz, Austria; marlies.hoermann-wallner@fh-joanneum.at; 7 Institute of Medical Sciences, School of Medicine, Medical Sciences and Nutrition, University of Aberdeen, Aberdeen AB25 2ZD, UK; christine.moelzer@abdn.ac.uk

**Keywords:** bilirubin, unconjugated bilirubin, oxidative stress, inflammation, antioxidants, FRAP

## Abstract

Bilirubin is an important antioxidant and a modulator of biological functions. However, most of the protection against oxidative stress was shown in vitro or ex vivo. The aim of this case-control study was to investigate whether subjects with Gilbert’s syndrome (GS) experience different levels of lipid and protein oxidation (as well as differences in oxidative stress related markers) compared to healthy controls. GS subjects (*n* = 119) demonstrated higher serum levels of unconjugated bilirubin (*p* < 0.001), a lower BMI (*p* < 0.001), 37% higher antioxidant potential assessed as ferric reducing ability potential (*p* < 0.001), higher advanced oxidation protein products (*p* < 0.01) andlower apolipoprotein B (*p* < 0.05), hs-C-reactive protein (*p* < 0.05), interleukin 6 (*p* < 0.001) and interleukin 1 beta (*p* < 0.05) values compared to healthy controls (*n* = 119). Furthermore, the resting heart rate was significantly lower in the GS group (*p* < 0.05). Stronger protective effects for GS subjects were demonstrated in the older subgroup (*n* = 104, average age 50 years) compared to those of the younger group (*n* = 134, average age 27 years). Although not all markers related to oxidative stress were different between the groups (e.g., malondialdehyde, homocysteine, oxLDL, and myeloperoxidase; *p* > 0.05), the observed differences contribute to the explanation of why GS serves as an important protector in the pathogenesis of metabolic, oxidative stress related diseases.

## 1. Introduction

Mild hyperbilirubinemia, also known as Gilbert’s syndrome (GS), is prevalent in 5–10% of the general population, up to 20% in some areas such as the Middle East [1]. This condition is based on various underlying promoter polymorphisms in the UDP glucuronosyltransferase 1A1 (UGT1A1) gene leading to a reduced conjugating activity of this enzyme, which is phenotypically resulting in a mild increase in unconjugated bilirubin (UCB) (total bilirubin >17.1 µM/L) [2].

Individuals with GS are protected from coronary artery disease (CAD), ischemic heart disease, atherosclerosis, all-cause mortality, and some cancers (e.g., lung cancer [3,4,5,6,7,8]). It has also been documented as playing a protective role against diabetes mellitus type 2 (DMT2) [9,10].

All latter chronic diseases are linked to oxidative stress, as reactive oxygen species (ROS) are involved in their pathogenesis. Furthermore, one thing that most of the chronic diseases have in common is that their development is not an immediate process, but usually requires years, triggered by an excessive intracellular increase in ROS [11]. Bilirubin is well established for its antioxidative potential, with most data generated in vitro or ex-vivo. For example, UCB was reported to possess antioxidant activity against peroxyl radicals [12] and was shown to function as a co-antioxidant with α-tocopherol to inhibit low-density lipoprotein cholesterol (LDL-C) oxidation, providing an explanation for how UCB may protect from atherosclerosis [13]. This antioxidant mediated protection was also seen through the inhibition of copper induced lipid peroxidation in individuals with GS [14]. Hyperbilirubinemia and liver heme oxygenase-1 (HO-1) induction in lipopolysaccharide (LPS)-treated rats resulted in a 2-fold accumulation of tissue UCB, which was associated with enhanced protection against lipid peroxidation [15].

In one of the few human studies, Maruhashi et al. reported lower levels of oxidative stress in GS subjects, mainly reduced serum concentrations of malondialdehyde (MDA)-modified LDL and urinary excretion of 8-hydroxy-2’-deoxyguanosine compared to controls and an enhanced endothelium-dependent vasodilation [16]. Vitek et al. observed a higher total antioxidant status in GS compared to healthy controls and patients with ischemic heart disease [17]. 

A very recent study showed a higher total antioxidant status in GS vs. controls, but exhibited no difference in the total oxidative status or the oxidative stress index [18].

Boon et al. reported that GS individuals possess reduced serum concentrations of oxidized LDL (oxLDL) and LDL-C [19]. The same group observed that UCB supplementation to serum/plasma could inhibit protein and lipid modification by quenching chloramines induced by myeloperoxidase (MPO)-induced hypochlorous assay (HOCl). The same was observed when investigating GS serum/plasma, which showed significantly reduced chloramine formation after MPO-induced oxidation [20].

Conversely, increased oxidative stress during hyperbilirubinemia has been reported in newborns before phototherapy. Here MDA and S100B protein levels were increased compared to control newborns. After the phototherapy advanced oxidation protein products (AOPP) and MDA levels decreased, suggesting that accumulating UCB may also be pro-oxidant [21].

Since human data were limited, the aims of this secondary analysis of two case-control studies were (i) to investigate whether GS subjects experience different levels of lipid and protein oxidation, (ii) to expand the analysis to oxidative stress related marker including blood pressure (BP) and resting heart rate and (iii) to assess whether the effects are depending on sex and age when compared to age and sex matched controls.

## 2. Materials and Methods

### 2.1. Study Population

A total of 238 subjects were included in this secondary evaluation of two case control trials [22,23]. ALT (alanine transferase), AST (aspartate aminotransferase), γ-GT (gamma-glutamyltransferase), LDH (lactate dehydrogenase), ALP (alkaline phosphatase), hemoglobin and hematocrit were measured at initial screening examinations. The exclusion criteria for these studies included age <20 years, pregnancy, chronic disease, alcoholism (>7 standard drinks/week), smoking (>1 cigarettes/day), excessive physical activity (>10 h/week), and the intake of any medication or supplements [23,24,25]. As an important diagnostic procedure, subjects were required to complete 400 kcal restricted fasting-protocol on the day preceding blood sampling, leading to increased serum UCB levels in the absence of liver disease [24]. The criterion for group allocation (GS or control group) was based on a fasting serum UCB concentration of ≥ or <17.1 µM/L, respectively. Both groups (*n* = 119 each) were matched for sex and age. Furthermore, the study population was divided into older and younger subsets (cut-off: 35 years).

All studies of this secondary evaluation had been approved by local ethical committees (274/2010, 1164/2014) [23,24] and were conducted in accordance with the Declaration of Helsinki. All participants provided signed informed consent.

### 2.2. Antropometric Measurements and Blood Pressure

Anthropometric measurements, BP, and the resting heart rate were obtained from participants who were barefoot and lightly dressed in the mornings of the study days. Body height was measured by stadiometer (model 214, Seca) to the nearest 0.5 cm and body weight using standard analogous scales (Selecta 791, Seca). Waist circumference (WC) and hip circumference (HC) were measured by tape (model 203, Seca). Body mass index (BMI) and waist-to-hip ratio (WHR) were calculated using the equations BMI = kg/m^2^ and WHR = WC/HC, respectively.

### 2.3. Blood Biochemistry

For each subject, an overnight fasting blood sample was collected into serum tubes. Samples were kept cool and protected from light until being analyzed or aliquoted (sample aliquots were stored at −80 °C for further analyses).

Serum UCB concentrations were analyzed following a well-established high performance liquid chromatography (HPLC) protocol [24,26]. Briefly, serum UCB concentrations were measured by HPLC (Merck, Hitachi, LaChrom), equipped with a Fortis C18 HPLC column (4.6 × 150 mm, 3 mm), a Phenomenex SecurityGuard cartridges for C18 HPLC columns (4 × 3 mm), and a photodiode array detector (PDA, Shimadzu) [24,26].

AST, ALT, GGT, LDH, apolipoprotein A1 (Apo-A1), apolipoprotein B (Apo-B), and uric acid were analyzed in the central laboratories of the Vienna General Hospital (Olympus 5400 clinical chemistry analyzers, Beckman Coulter) on the day of blood sampling.

Interleukin 6 (IL-6) and Interleukin 1 beta (IL-1ß) levels were measured with high-sensitive ELISA (eBioscience) [22] as well as determined in monocytes (CD14 ^+^ ) following the standard intracellular protein staining protocol (BD) using a FACS Calibur (BD) flow cytometer [27].

MPO was measured using the immunodiagnostic MPO enzyme-linked immunosorbent assay (ELISA) kit (Immundiagnostik AG, Bensheim, Germany). 

High-sensitivity -C-reactive protein (hs-CRP) was analyzed using the hs- CRP Latex immune–turbidimetric assay (Olympus 5400 clinical chemistry analyzers, Beckman Coulter).

MDA levels were determined in plasma as described previously [28]. After heating (60 min, 100 °C), plasma samples were neutralized with methanol/NaOH, centrifuged (3 min, 3000 rpm) and their MDA was measured with HPLC (excitation: λ 532 nm, emission: λ 563 nm, LaChrom Merck Hitachi Chromatography System, Vienna, Austria; HPLC column 125 × 4 mm, 5 μm; Merck, Vienna, Austria).

The antioxidant capacity of serum was measured via the ferric reducing ability potential (FRAP) assay as described earlier [28] in triplicates, using trolox as a standard. Absorbance was measured with BMG FLUOstar OPTIMA Microplate Reader (BMG LABTECH GmbH) at 593 nm and results are expressed as trolox equivalents in μmol/L.

oxLDL concentrations were measured using an ELISA kit (Mercodia AB, Uppsala, Sweden). AOPP were determined via a colorimetric assay kit (Immundiagnostik AG, Bensheim, Germany). For both oxLDL and AOPP, the absorbance of samples and standards were read with a Fluostar Optima microplate reader (BMG labtechnologies, Germany), and all measures were made in duplicate.

Glutathione (GSH) was determined after erythrocyte release photometrically, as described earlier [29].

Plasma homocysteine was determined using HPLC as described elsewhere [30], with a fluorescence detector (emission wavelength: 515 nm; excitation wavelength: 385 nm) on a RP LiChrosphere column (5 µm, 125 × 4 mm) (Merck, Hitachi, LaChrom, Austria). A potassium hydrogenphosphate buffer including 4% acetonitrile was used as mobile phase.

### 2.4. Statistical Analysis

Statistical analyses were performed using SPSS (IBM statistics, Version 26.0). Prior to analysis, missing values had been excluded. A *p* < 0.05 was considered significant for all procedures. The Kolmogorov Smirnov test was used to determine data distribution. For comparison of two groups, the independent samples t-test (parametric data) or Mann–Whitney U test (non-parametric data) were used. For IL-6 and IL-1ß data the z-score was calculated and is presented for comparison of the different interleukin measurement methods used in the two studies. Correlation between variables were analyzed by Pearson or Spearman correlation.

As serum bilirubin levels are physiologically higher in men than they are in women, we decided a priori to run all models separately for men and women. Additionally, the age associated effects related to mild hyperbilirubinaemia were observed in previous studies in respect to indicators of metabolic health in men and women [22,31], so we tested for effect modification by categories of age in our study as well.

## 3. Results

### 3.1. Gilbert’s Syndrome vs. Control Group

The GS group demonstrated higher serum UCB levels (30.8 ± 11.6 vs. 8.6 ± 3.8, *p* < 0.001) and a lower BMI (−8%, *p* < 0.001) compared to the control group. There was no difference in the assessed liver enzyme activities. GS subjects experienced 37% higher FRAP values (688 ± 184 vs. 504 ± 114 *p* < 0.001), higher AOPP (46.5 ± 16.5 vs. 37.2 ± 11.8, *p* < 0.01) and lower Apo-B (84.1 ± 23.6 vs. 91.0 ± 24.3 *p* < 0.05), hs-CRP (0.08 ± 0.09 vs. 0.13 ± 0.16 *p* < 0.05), IL-6 (−0.31 ± 0.80 vs. 0.30 ± 1.08 *p* < 0.001) and IL-1ß (−0.21 ± 0.96 vs. 0.21 ± 1.00 *p* < 0.05) values.

Furthermore, the resting heart rate was significantly lower in the GS group (70.2 ± 11.3 vs. 75.0 ± 11.0 *p* < 0.05).

All other parameters did not differ between GS subjects and the control group (Table 1).

### 3.2. Gilbert’s Syndrome vs. Control Group, in Two Age Subgroups (</≥35 Years)

GS individuals within the younger subgroup (*n* = 134, average age of 27 years), showed beside higher UCB levels, elevated FRAP (686 ± 196 vs. 511 ± 106, *p* < 0.001) and AOPP (50.1 ± 17.2 vs. 37.6 ± 12.2, *p* < 0.01) values, but lower IL-6 (−0.37 ± 0.90 vs. 0.34 ± 1.04, *p* <0.001) and a tendency for lower hs-CRP levels (0.07 ± 0.06 vs. 0.12 ± 0.17, *p* = 0.055) compared to the control group.

Within the older subgroup (*n* = 104, average age of 50 years) that is at higher risk for chronic diseases, the GS group had a 13% lower BMI (23.6 ± 3.14 vs. 27.1 ± 4.55, *p* < 0.001), significantly higher UCB levels (31.1 ± 12.3 vs. 7.55 ± 3.5, *p* < 0.001) and 28% higher FRAP values (692 ± 167 vs. 495 ± 126, *p* < 0.001). However, at the same time, they showed significantly lower Apo-B (86.7 ± 25.1 vs. 104 ± 22.1 *p* < 0.001, −17%), Apo-B:Apo-A1 ratio (0.58 ± 0.22 vs. 0.72 ± 0.23, *p* < 0.01), serum amyloid A (SAA, 4.33 ± 1.52 vs. 5.44 ± 2.65, *p* < 0.05), IL-6 (−0.25 ± 0.88 vs. 0.31 ± 0.96, *p* < 0.05), and resting heart rate (67.9 ± 11.1 vs. 75.4 ± 12.1, *p* < 0.05) compared to controls.

All further oxidative stress markers did not show significant differences (Table 2).

### 3.3. Gilbert’s Syndrome vs. Control Group, Sex Specific Differences

GS participants (males and females) reported higher UCB serum levels than the controls (*p* < 0.001) and a lower BMI (males 6% lower in GS group, *p* < 0.05; females 10% lower in GS group vs. controls, *p* < 0.01) (Table 3). Similar to the other groups, FRAP values were higher in GS subjects of both sexes (males: 709 ± 192 vs. 519 ± 89 *p* < 0.001; females: 643 ± 159 vs. 471 ± 152 *p* < 0.001).

All other differences were sex specific. Male GS subjects showed higher AOPP (50.6 ± 16.0 vs. 39.6 ± 10.9, *p* < 0.01), lower IL-6 (−0.32 ± 0.72 vs. 0.31 ± 1.08 *p* < 0.001) and IL-1ß (−0.25 ± 0.84 vs. 0.47 ± 0.61 *p* < 0.01) values compared to controls. Male GS subjects tended to have a lower oxLDL:LDL ratio (*p* = 0.073) and higher MPO values (*p* = 0.080).

Female GS participants had a higher oxLDL:LDL ratio (3.18 ± 3.72 vs. 1.18 ± 1.51 *p* < 0.05) but lower Apo-B (76.6 ± 16.6 vs. 90.0 ± 19.5 *p* < 0.01), SAA (4.03 ± 1.14 vs. 5.36 ± 2.58 *p* < 0.05), hs-CRP (0.07 ± 0.08 vs. 0.16 ± 0.21 *p* < 0.05) and resting heart rate (67.2 ± 10.6 vs. 76.3 ± 9.8 *p* < 0.05). Furthermore, they showed a trend for lower Apo-B:Apo-A1 ratio (*p* = 0.090) and lower IL-6 (*p* = 0.065).

### 3.4. Correlations of UCB Concentrations with Oxidative Stress Marker, Sex Specific Differences

UCB concentrations showed in the total study group strong correlations with FRAP (r = 0.601, *p* < 0.01) and weaker, but significant correlations with BMI, hs-CRP, IL-6 (all *p* < 0.01), Apo B, the Apo-B:Apo-A1 ratio, IL-1ß and homocysteine (all *p* < 0.05) (Table 4).

When dividing the group into the younger and older subgroups, stronger associations can be observed in the older age subgroup (Table 5).

In the younger subgroup strong correlations with UCB were found for FRAP (r = 0.654, *p* < 0.01) and homocysteine (r = 0.283, *p* < 0.01) and weaker negative associations ones for hs-CRP (*p* < 0.05) and IL-6 (*p* < 0.05).

In the older age subgroup, associations were more frequent and stronger (e.g., FRAP: r = 0.654, *p* < 0.01; GSH: r = −0.551, *p* < 0.01; BMI: r = −0.401, *p* < 0.01, see Table 5).

## 4. Discussion

The aim of this secondary evaluation of two case control studies was to investigate whether subjects with GS, who show higher UCB serum levels than the population, experience lower oxidative stress and oxidative stress related biomarkers. A total of 238 sex and age matched subjects were included, 65% males and 35% females, which reflects the well-established higher prevalence of GS in males [32].

As already shown by us and others [31,33], the BMI in the GS group, independently of sex, was significantly lower. This was even more pronounced in the older subgroup, which showed a 13% lower BMI than the control group. This is important for metabolic health, since body weight (and being overweight and obese in particular) are risk factors for various chronic diseases [34,35]. Since body weight and composition is changing with age [36] the GS phenotype with a lower body weight in the 4th to 6th decade of life compared to controls, significantly contribute to the reduced risk of cardiovascular disease (CVD) and all-cause mortality in this population group [37].

We investigated the oxidation products MDA, oxLDL, AOPP, the total antioxidant capacity (FRAP), antioxidants such as GSH or uric acid, inflammatory markers (mainly IL-6, IL-1ß, hs-CRP or MPO) and other biochemical biomarkers which are associated risk markers for CVD (such as homocysteine, Apo-A1 and Apo-B).

There was no difference in MDA and oxLDL levels between the groups, independently of sex and age. FRAP values were significantly higher in the GS group and all subgroups indicating a higher total antioxidant capacity in the plasma of mildly hyperbilirubinaemic adults, which might be explained by their higher UCB concentrations. This equips the blood with a higher resistance against ROS. A similar outcome was shown by Copur et al. [18], who also observed an increased total antioxidant status in GS subjects. Contrarily, AOPP, a marker for protein oxidation, was increased in the GS group. This was mainly based on differences in the young subgroup and male GS subjects, whereas no difference was found in females and the older subgroup. This observation was consistent with a study of newborns with high bilirubin levels, which showed no difference in AOPP levels when compared to normobilirubinemic newborns [21] and different to Boon et al., who supplemented plasma/serum with exogenous UCB and observed an inhibition of protein carbonyls [20]. Otherwise, clinical data on protein oxidation are missing.

Uric acid and GSH were not different between groups. Uric acid is similar to bilirubin (another important endogenous antioxidant in blood) and seems to be not affected by the GS condition, despite the chronically increased bilirubin concentrations in the blood. The same is true for GSH, which is negatively correlated with UCB in our study. Boon et al. showed in a smaller human study higher GSH levels in GS [19]. In the same study, they obtained lower oxLDL levels in GS subjects compared to controls. Regarding oxLDL, we did not observe significant differences. However, mean oxLDL levels were 29% lower in GS subjects and even 36% lower in GS subjects of the older subgroup compared to controls, which is of biological relevance. Interestingly a sex specific effect is demonstrated regarding oxLDL in our study. Male GS subjects had 52% lower oxLDL levels (*p* = 0.090), but female GS 50% increased oxLDL values compared to the controls. No correlation between oxLDL and UCB was observed. However, a strong negative association between UCB and GSH exclusively in the older age subgroup (r = −0.551, *p* < 0.01) was revealed.

MPO a heme-containing peroxidase catalyzes the formation of reactive oxygen intermediates, including HOCl, which reacts with most biological molecules. MPO was always higher in GS groups, although the difference never reached statistical significance. An interesting observation is that MPO as well as AOPP showed a tendency to be higher only in male GS subjects and in the younger GS subgroup.

Biomarkers to describe the CVD risk include the structural proteins of lipoproteins. Apo-A1 and A-2 are the major structural proteins of high-density lipoprotein cholesterol (HDL-C) particles, whereas Apo-B is a major protein of every other lipoprotein particle except HDL-C. The Apo-B/Apo-A1 ratio is considered as one of the strongest plasma lipid-associated predictors of CVD risk [38], which indicates the balance between potentially atherogenic and anti-atherogenic particles [39]. GS subjects showed similar Apo-A1 concentrations but lower Apo-B concentrations than control subjects (Table 1). This was mainly driven by the older subgroup with significantly lower Apo-B levels (17% lower in GS), which is reflected in the significantly lower Apo-B:Apo-A1 ratio (*p* < 0.01, Table 2). Differences in apolipoproteins might also be sex-specific since significantly lower Apo-B levels and Apo-B:Apo-A1 ratios were exclusively observed in females. (Table 3). Similarly, SAA, a proinflammatory adipokine in humans linked to obesity and a predictor of CVD [40,41] was significantly lower (*p* < 0.05, Table 2 and Table 3) in female GS participants and the older GS subgroup. This also has health impact, since recently SAA levels are associated with all-cause mortality in women with signs and symptoms of ischemia, nonobstructive CAD and preserved left ventricular ejection fraction [42].

Further proinflammatory markers such as hs-CRP, IL-6 and IL-1ß were significantly lower in the GS group. This shows again that the risk reduction of GS against chronic diseases is multifactorial as these marker are related to a lower risk of CVD [43] and DMT2 [44].

A high heart rate is associated with a higher risk of all-cause mortality and cardiovascular events [45] and high BP is one of the most important risk factors for CVD [46] which is the leading cause of mortality. Approximately 54% of strokes and 47% of coronary heart diseases worldwide are attributable to high BP [47]. Therefore, we were interested in whether the GS condition might have an impact on these risk factors. Although systolic and diastolic BP was always marginally lower in the GS groups, no statistical significance was observed. However, the resting heart rate was significantly lower, on average by five beats per minute (bpm). Similar to other biomarkers discussed before, there was no difference in males and the young subgroup. However, GS females and the older subgroup showed 9 and 7 bpm difference respectively, less than the controls. This is a significant health outcome, since it has been shown that an increase in heart rate by 10 bpm was associated with an increase in the risk of cardiac death by at least 20%, and this increase in the risk is similar to the one observed with an increase in systolic BP by 10 mm Hg [45].

This study has strengths and limitations. One strength is the high number of subjects obtained by pooling the data of two studies together, which gave us the possibility to also have appropriate numbers for the subgroup analysis (age and sex). Furthermore, we were able to consider data for lipid and protein oxidation as well as inflammation and other endogenous antioxidants to give a comprehensive dataset. Bilirubin was assessed as UCB with HPLC and not as total bilirubin with a diazo method, as usually performed in the clinical and pre-clinical setting. One limitation of the study is that only the BMI is shown and not body composition data. Furthermore, the age range of the investigated group was young to old adults with less participants aged 65 and older. The amount of missing data in some variables is a general limitation of observational datasets.

## 5. Conclusions

GS subjects showed increased antioxidant status (FRAP) and oxidized proteins (AOPP) at the same time, and they demonstrated reduced Apo-B, hs-CRP, IL-6 and IL-1ß, which are pro-oxidant/proinflammatory markers. Furthermore, the resting heart rate and the BMI was lower compared to healthy controls.

A stronger protective effect was demonstrated for GS participants in their 4–6th decade of life and for females in all age subgroups. Since most of the parameters investigated contribute to the pathogenesis of chronic disease, the presented data contribute to the explanation as to why GS subjects have a reduced risk for many chronic diseases.

## Figures and Tables

**Table 1:** Demographic features and biomarkers for oxidative stress and inflammation of individuals with GS (*n* = 119) and controls (*n* = 119).

Parameters	Controls	Gilbert’s Syndrome	*p*-Value
Age (years)	37.0 (13.7)	37.0 (13.7)	0.989
BMI (kg/m^2^)	24.9 (4.37)	23.0 (3.10)	**<0.001**
UCB (µM/L)	8.64 (3.82)	30.8 (11.6)	**<0.001**
γ-GT (U/L)	22.2 (13.6)	24.4 (25.9)	0.416
AST (U/L)	25.3 (7.86)	27.0 (8.85)	0.127
ALT (U/L)	22.6 (9.42)	23.7 (11.2)	0.408
Homocysteine (µM/L)	11.1 (3.89)	11.6 (4.61)	0.457
Uric acid (mg/dl)	5.31 (1.13)	5.48 (1.12)	0.280
MDA (µM/L)	1.37 (0.66)	1.38 (0.62)	0.956
FRAP (µM/L)	504 (114)	688 (184)	**<0.001**
oxLDL (ng/mL)	248 (481)	176 (226)	0.298
oxLDL:LDL ratio	2.54 (4.34)	2.13 (2.82)	0.547
GSH (mg/dl)	74.2 (9.02)	72.0 (11.0)	0.329
AOPP (µM/L)	37.2 (11.8)	46.5 (16.5)	**0.006**
Apo-A1 (mg/dl)	147 (25.1)	147 (23.6)	0.881
Apo-B (mg/dl)	91.0 (24.3)	84.1 (23.6)	**0.029**
Apo-B:Apo-A1 ratio	0.63 (0.22)	0.58 (0.19)	0.077
SAA (mg/l)	4.66 (2.14)	4.44 (3.94)	0.639
MPO (µM/L)	48.0 (16.1)	56.8 (23.8)	0.073
hs-CRP (mg/dl)	0.13 (0.16)	0.08 (0.09)	**0.017**
IL-6 *	0.31 (1.08)	−0.31 (0.80)	**<0.001**
IL-1ß *	0.21 (1.00)	−0.21 (0.96)	**0.023**
Resting heart rate (bpm)	75.0 (11.0)	70.2 (11.3)	**0.032**
Systolic BP (mmHg)	133 (15.7)	130 (13.0)	0.318
Diastolic BP (mmHg)	69.1 (11.9)	67.4 (11.9)	0.419

Abbreviation: BMI (body mass index), UCB (unconjugated bilirubin), γ-GT (gamma-glutamyltransferase), AST (aspartate aminotransferase), ALT (alanine transferase), MDA (malondialdehyde), FRAP (ferric reducing ability potential), oxLDL (oxidized LDL), GSH (glutathione), AOPP (advanced oxidation protein products), Apo-A1 (apolipoprotein A1), Apo-B (apolipoprotein B), SAA (serum amyloid A), MPO (myeloperoxidase), hs-CRP (high-sensitive C-reactive protein), IL-6 (interleukin 6), IL-1ß (interleukin 1 beta), BP (blood pressure). * Used standardized scores (z-scores) to compare different measurements in the two studies. Bold *p*-values indicate statistically significant differences between the groups.

**Table 2:** Demographic features, biochemical parameters, biomarkers for oxidative stress and inflammation of individuals with GS and controls in two age subgroups.

	Age < 35 Years (*n* = 134)			Age ≥ 35 Years (*n* = 104)		
Parameters	Controls	Gilbert’s Syndrome	*p*-Value	Controls	Gilbert’s Syndrome	*p*-Value
Age (years)	26.6 (3.9)	26.7 (3.8)	0.887	49.9 (10.0)	50.2 (10.0)	0.846
BMI (kg/m^2^)	23.3 (3.9)	22.6 (3.8)	0.213	27.1 (4.55)	23.6 (3.14)	**<0.001**
UCB (µM/L)	9.52 (3.89)	30.5 (11.2)	**<0.001**	7.55 (3.45)	31.1 (12.3)	**<0.001**
γ-GT (U/L)	17.8 (7.83)	22.7 (30.0)	0.214	27.6 (16.9)	26.7 (19.3)	0.818
AST (U/L)	25.7 (8.61)	26.6 (8.30)	0.585	24.9 (6.91)	27.6 (9.56)	0.094
ALT (U/L)	22.5 (9.29)	23.5 (11.7)	0.591	22.7 (9.67)	23.9 (10.6)	0.521
Homocysteine (µM/L)	10.9 (4.07)	12.3 (5.46)	0.105	11.5 (3.63)	10.4 (2.44)	0.127
Uric acid (mg/dl)	5.33 (1.11)	5.62 (1.14)	0.165	5.28 (1.16)	5.27 (1.07)	0.973
MDA (µM/L)	1.30 (0.63)	1.34 (0.65)	0.710	1.48 (0.69)	1.43 (0.58)	0.725
FRAP (µM/L)	511 (106)	686 (196)	**<0.001**	495 (126)	692 (167)	**<0.001**
oxLDL (ng/mL)	217 (296)	170 (205)	0.461	285 (643)	183 (252)	0.445
oxLDL:LDL ratio	2.96 (4.57)	1.98 (2.64)	0.304	2.03 (4.06)	2.30 (3.04)	0.779
GSH (mg/dl)	71.6 (8.39)	71.7 (11.3)	0.992	80.0 (7.57)	72.8 (10.7)	0.081
AOPP (µM/L)	37.6 (12.3)	50.1 (17.2)	**0.004**	36.5 (11.1)	37.6 (10.7)	0.391
Apo-A1 (mg/dl)	146 (27.7)	143 (24.7)	0.432	150 (21.4)	153 (20.9)	0.432
Apo-B (mg/dl)	80.9 (21.1)	81.9 (22.3)	0.781	104 (22.1)	86.7 (25.1)	**<0.001**
Apo-B:Apo-A1 ratio	0.58 (0.19)	0.58 (0.18)	0.889	0.72 (0.23)	0.58 (0.22)	**0.009**
SAA (mg/l)	4.11 (1.48)	4.52 (4.96)	0.553	5.44 (2.65)	4.33 (1.52)	**0.027**
MPO (µM/L)	44.4 (12.8)	52.2 (19.1)	0.094	57.1 (20.2)	70.2 (31.5)	0.288
hs-CRP (mg/dl)	0.12 (0.17)	0.07 (0.06)	0.055	0.15 (0.16)	0.10 (0.13)	0.131
IL-6 *	0.34 (1.04)	−0.37 (0.90)	**0.001**	0.31 (0.96)	−0.25 (0.88)	**0.012**
IL-1ß *	0.20 (0.89)	−0.15 (0.85)	0.116	0.22 (1.14)	−0.28 (1.09)	0.106
Resting heart rate (bpm)	74.8 (10.5)	71.9 (11.4)	0.309	75.4 (12.1)	67.9 (11.1)	**0.045**
Systolic BP (mmHg)	131 (16.7)	129 (12.7)	0.571	131 (16.7)	129 (12.7)	0.405
Diastolic BP (mmHg)	66.6 (13.1)	66.1 (9.6)	0.849	66.6 (13.1)	66.1 (9.56)	0.359

Abbreviation: BMI (body mass index), UCB (unconjugated bilirubin), γ-GT (gamma-glutamyltransferase), AST (aspartate aminotransferase), ALT (alanine transferase), MDA (malondialdehyde), FRAP (ferric reducing ability potential), oxLDL (oxidized LDL), GSH (glutathione), AOPP (advanced oxidation protein products), Apo-A1 (apolipoprotein A1), Apo-B (apolipoprotein B), SAA (serum amyloid A), MPO (myeloperoxidase), hs-CRP (high-sensitive C-reactive protein), IL-6 (interleukin 6), IL-1ß (interleukin 1 beta), BP (blood pressure). * Used standardized scores (z-scores) to compare different measurements in the two studies. Bold *p*-values indicate statistically significant differences between the groups.

**Table 3:** Demographic features, biochemical parameters, and biomarkers for oxidative stress and inflammation of individuals with GS and controls according to sex.

	Males (*n* = 154)			Females (*n* = 84)		
Parameters	Controls	Gilbert’s Syndrome	*p*-Value	Controls	Gilbert’s Syndrome	*p*-Value
Age (years)	35.8 (13.7)	35.2 (13.4)	0.797	39.2 (13.7)	40.2 (13.9)	0.737
BMI (kg/m^2^)	22.3 (4.5)	23.7 (3.2)	**0.010**	24.1 (4.31)	21.7 (2.45) ^a^	**0.002**
UCB (µM/L)	9.53 (3.75)	33.2 (16.6)	**<0.001**	6.95 (3.38) ^b^	26.4 (8.01) ^a^	**<0.001**
γ-GT (U/L)	23.7 (10.0)	28.0 (30.5)	0.268	19.4 (14.5)	17.8 (11.5) ^a^	0.632
AST (U/L)	27.0 (7.73)	28.3 (8.44)	0.336	22.1 (7.11) ^b^	24.6 (9.17) ^a^	0.165
ALT (U/L)	24.9 (8.78)	25.9 (11.5)	0.542	18.2 (9.11) ^b^	19.6 (9.33) ^a^	0.471
Homocysteine (µM/L)	12.0 (4.17)	12.3 (4.61)	0.747	9.08 (2.10) ^b^	10.0 (4.24) ^a^	0.315
Uric acid (mg/dl)	5.79 (0.90)	5.92 (0.88)	0.377	4.22 (0.78) ^b^	4.48 (0.96) ^a^	0.254
MDA (µM/L)	1.28 (0.63)	1.38 (0.63)	0.371	1.59 (0.67) ^b^	1.37 (0.61) ^a^	0.208
FRAP (µM/L)	519 (89)	709 (192)	**<0.001**	471 (152)	643 (159) ^a^	**<0.001**
oxLDL (ng/mL)	317 (571)	154 (183)	0.090	109 (135)^b^	219 (294) ^a^	0.137
oxLDL:LDL ratio	3.22 (5.09)	1.61 (2.12)	0.073	1.18 (1.51)	3.18 (3.72) ^a^	**0.032**
GSH (mg/dl)	73.4 (8.57)	70.0 (11.4)	0.213	76.7 (10.23)	77.6 7.98)	0.836
AOPP (µM/L)	39.6 (10.9)	50.6 (16.0)	**0.004**	30.5 (12.1)	35.0 (12.3) ^a^	0.423
Apo-A1 (mg/dl)	139 (20.0)	139 (20.8)	0.953	165 (25.4) ^b^	162 (21.0) ^a^	0.553
Apo-B (mg/dl)	91.5 (26.6)	88.1 (25.8)	0.422	90.0 (19.5)	76.6 (16.6) ^a^	**0.001**
Apo-B:Apo-A1 ratio	0.67 (0.23)	0.62 (0.19)	0.242	0.55 (0.17) ^b^	0.47 (0.14) ^a^	0.090
SAA (mg/l)	4.37 (1.86)	4.62 (4.67)	0.674	5.36 (2.58) ^b^	4.03 (1.14)	**0.014**
MPO (µM/L)	47.5 (16.6)	58.4 (25.6)	0.080	49.1 (15.6) ^b^	52.3 (18.2)	0.682
hs-CRP (mg/dl)	0.12 (0.14)	0.09 (0.10)	0.202	0.16 (0.21)	0.07 (0.08)	**0.033**
IL-6 *	0.31 (1.08)	−0.32 (0.72)	**<0.001**	0.34 (1.37)	−0.27 (0.98)	0.065
IL-1ß *	0.47 (0.61)	−0.25 (0.84)	**0.002**	−0.25 (0.75) ^b^	−0.15 (1.20)	0.771
Resting heart rate (bpm)	74.2 (11.8)	71.5 (11.6)	0.354	76.3 (9.8)	67.2 (10.6)	**0.012**
Systolic BP (mmHg)	136 (15.0)	133 (11.5)	0.367	126 (15.4) ^b^	123 (13.9) ^a^	0.515
Diastolic BP (mmHg)	71.3 (11.7)	70.3 (10.9)	0.685	64.8 (11.4) ^b^	60.9 (12.0) ^a^	0.326

Abbreviation: BMI (body mass index), UCB (unconjugated bilirubin), γ-GT (gamma-glutamyltransferase), AST (aspartate aminotransferase), ALT (alanine transferase), MDA (malondialdehyde), FRAP (ferric reducing ability potential), oxLDL (oxidized LDL), GSH (glutathione), AOPP (advanced oxidation protein products), Apo-A1 (apolipoprotein A1), Apo-B (apolipoprotein B), SAA (serum amyloid A), MPO (myeloperoxidase), hs-CRP (high-sensitive C-reactive protein), IL-6 (interleukin 6), IL-1ß (interleukin 1 beta), BP (blood pressure). ^a^ Females with GS different to males with GS, *p* < 0.05, ^b^ Female control different to male control, *p* < 0.05. * Used standardized scores (z-scores) to compare different measurements in the two studies. Bold *p*-values indicate statistically significant differences between the groups.

**Table 4:** Correlations between UCB and oxidative stress related marker in the total study group.

Parameters	r	*p*-Value
BMI	−0.248	<0.01
Apo-B	−0.152	<0.05
Apo-B:Apo-A1 ratio	−0.153	<0.05
hs-CRP	−0.192	<0.01
IL-6	−0.301	<0.01
IL-1ß	−0.211	<0.05
Homocysteine	0.151	<0.05
FRAP	0.601	<0.01

Abbreviation: BMI (body mass index), Apo-B (apolipoprotein B), Apo-A1 (apolipoprotein A1), hs-CRP (high-sensitive C-reactive protein), IL-6 (interleukin 6), IL-1ß (interleukin 1 beta), FRAP (ferric reducing ability potential).

**Table 5:** Correlations with UCB concentrations in age subgroups.

	Age <35 Years			Age ≥35 Years	
Parameters	r	*p*-Value	Parameters	r	*p*-Value
hs-CRP	−0.199	<0.05	BMI	−0.401	<0.01
IL-6	−0.296	<0.05	Apo-B	−0.261	<0.01
Homocysteine	0.283	<0.01	Apo-B:Apo-A1	−0.267	<0.01
FRAP	0.654	<0.01	Homocysteine	−0.296	<0.05
			SAA	−0.253	<0.05
			GSH	−0.551	<0.01
			IL-6	−0.308	<0.05
			FRAP	0.654	<0.01

Abbreviation: hs-CRP (high-sensitive C-reactive protein), IL-6 (interleukin 6), FRAP (ferric reducing ability potential), BMI (body mass index), Apo-B (apolipoprotein B), Apo-A1 (apolipoprotein A1), SAA (serum amyloid A), GSH (glutathione), FRAP (ferric reducing ability potential).

## Data Availability

The data presented in this study are available in this article.

## Abbreviations

AOPP	advances oxidation protein products
APO	apolipoprotein
BMI	body mass index
BP	blood pressure
BPM	beats per minute
CVD	cardiovascular disease
FRAP	ferric reducing ability potential
GSH	glutathione
HDL-C	high-density lipoprotein cholesterol
HO-1	heme oxygenase 1
HPLC	high-performance liquid chromatography
hs-CRP	high-sensitive C-reactive protein
GS	Gilbert’s syndrome
IL	interleukine
LDL-C	low-density lipoprotein cholesterol
MDA	malondialdehyd
MPO	myeloperoxidase
oxLDL	oxidised low density lipoprotein
ROS	reactive oxygen species
SAA	serum amyloid A
UCB	unconjugated bilirubin
UGT1A1	uridine diphosphoglucuronyltransferase 1A1
